# Current Therapeutic Strategies for Adipose Tissue Defects/Repair Using Engineered Biomaterials and Biomolecule Formulations

**DOI:** 10.3389/fphar.2018.00507

**Published:** 2018-05-17

**Authors:** Christopher M. Mahoney, Cayla Imbarlina, Cecelia C. Yates, Kacey G. Marra

**Affiliations:** ^1^Department of Bioengineering, University of Pittsburgh, Pittsburgh, PA, United States; ^2^Department of Biology, Carlow University, Pittsburgh, PA, United States; ^3^Department of Pathology, University of Pittsburgh, Pittsburgh, PA, United States; ^4^Department of Health Promotion and Development, School of Nursing, University of Pittsburgh, Pittsburgh, PA, United States; ^5^McGowan Institute for Regenerative Medicine, Pittsburgh, PA, United States; ^6^Department of Plastic Surgery, University of Pittsburgh, Pittsburgh, PA, United States

**Keywords:** microspheres, adipose-derived stem cells, soft tissue defects, adipose tissue restoration, adipogenic factors

## Abstract

Tissue engineered scaffolds for adipose restoration/repair has significantly evolved in recent years. Patients requiring soft tissue reconstruction, caused by defects or pathology, require biomaterials that will restore void volume with new functional tissue. The gold standard of autologous fat grafting (AFG) is not a reliable option. This review focuses on the latest therapeutic strategies for the treatment of adipose tissue defects using biomolecule formulations and delivery, and specifically engineered biomaterials. Additionally, the clinical need for reliable off-the-shelf therapies, animal models, and challenges facing current technologies are discussed.

## Introduction

Adipose tissue is distributed throughout the body and is categorized by location such as subcutaneous (arm, abdominal, and gluteal), intraabdominal (omental, retroperitoneal, and visceral) and other sites (retroorbital, periarticular regions, bone marrow, intramuscular, and pericardial). Adipose tissue plays an essential role in the protection of underlying structures, providing insulation, contributing function, and imparting a normal human appearance. This appearance, and even function, can be compromised by disease, trauma, tumor resection, or congenital defects of the subcutaneous adipose tissue. These pathologies and defects of the adipose tissue remain a reconstructive challenge for plastic surgeons and clinicians. According to the American Society of Plastic Surgeons, the most common reconstructive procedures are breast construction, burn care, cleft lip/palate repair, tumor removal, and treatment from dog bites (ASPS, 2017). In particular, from 2009 to 2014, the Agency for Healthcare Research and Quality reported an increase in breast reconstruction after mastectomy by 62 percent (Miller et al., 2017).

Two major reconstruction options for breast adipose tissue deficits in the clinic include implant or flap surgery using the patient’s own skin, fat, and/or muscle. Currently, flap surgeries are not without morbidity. Patients receiving autologous reconstruction are at high risk for specific complications including wound infection, flap/prosthesis failure, and reoperation. Other general complications of major surgery such as deep vein thrombosis, blood transfusions, pneumonia, and pulmonary embolism can lead to prolonged length of stay in the hospital (Tachi and Yamada, 2005; Agha et al., 2013). Patients undergoing implant-based reconstruction may encounter complications such as rupture, migration, discontent, implant exposure/extrusion, rippling, and deformation/distortion (Rocco et al., 2016). With the volume of complications to consider using these invasive procedures, clinicians and patients are looking for less or minimally invasive options.

In effort to reduce the risk of complications, clinicians have increased the use of a widely used procedure in plastic surgery known as autologous fat grafting (AFG). Assuming the patient has adipose tissue to spare, AFG harvests the patient’s own fat, typically from the abdomen or thighs, via liposuction and deposits the tissue into the defect site with syringe injections. AFG is usually minimally invasive and carries several advantages in comparison to implant- or flap-based soft tissue reconstruction. With minimal scarring from the defect site and no foreign body reaction, the patient recovery time is less than 48 h. However, while the general outcomes for these procedures are positive in the short-term, the long-term results can be unpredictable due to post-graft resorption rates reaching as high as 90% (Hong et al., 2010; Monfort and Izeta, 2012). Another minimally invasive technique used for contour defects incorporates *in vitro* cultured adipocytes via injection (Kim et al., 2011). Unfortunately, this procedure’s efficacy is limited to eyelid wrinkles, deep nasolabial folds, depressed scars, and less projected forehead (Gir et al., 2012b; Monfort and Izeta, 2012).

The field of adipose tissue engineering evolved to address the clinical need discussed earlier. The following sections describe how current therapies insufficiently restore body contour. This review focuses on the wide range of biomaterials for resolving adipose tissue defects and the most current cutting edge therapeutic strategies, specifically encapsulated biomolecules. This comprehensive overview guides the rationale for the need of a reliable off-the-shelf biomaterial that will mostly likely contain a scaffold-encapsulation therapy combination to restore and repair the adipose defect site.

## Bioactive Molecules for Adipose Tissue Repair

**FIGURE 1:**
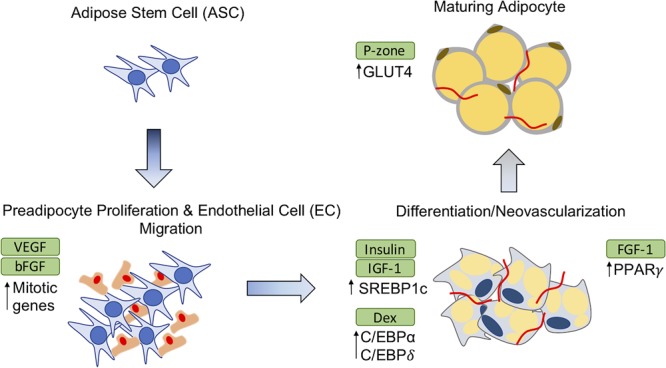
Effects of therapeutic agents on adipose-derived stem cell (ASC) differentiation. The addition of exogenous agents in adipose restoration therapies affect precursor adipocytes and maturing adipocytes in different ways to induce adipogenesis or vascularization. GLUT4, Glucose transporter; C/EBP, CCAT enhanced binding protein; SREBP1c, sterol regulatory element-binding protein-1c; FGF-2, Fibroblast growth factor-2; FGF-1, Fibroblast growth factor-1; P-zone, Pioglitazone hydrochloride; EC, endothelial cell; PPAR, peroxisome proliferator activated receptor; VEGF, vascular endothelial growth factor; Dex, Dexamethasone; IGF-1, insulin-like growth factor-1.

An increasingly widespread trend for adipose restoration has been to incorporate additional biomolecules or biological factors with scaffold material to facilitate new adipose tissue formation, thus creating composite biomaterials. Multi-component materials are becoming a common approach in recent years producing interesting results. Basic fibroblast growth factor-2 (FGF-2) (Kawaguchi et al., 1998; Kimura et al., 2003; Vashi et al., 2006; Zhang et al., 2016), fibroblast growth factor-1 (FGF-1) (Moya et al., 2010), dexamethasone (Rubin et al., 2009; Sun et al., 2013; Fan et al., 2015; Jia et al., 2015; Kelmendi-Doko et al., 2017), adipose-derived stem cells (ASCs) (Wang et al., 2013; Cheung et al., 2014; Brown et al., 2015), pioglitazone (Yazawa et al., 2015), insulin (Masuda et al., 2004; Rubin et al., 2009; Hong et al., 2010), and insulin-like growth factor-1 (IGF-1) (Masuda et al., 2004) are among several additive components that have been evaluated *in vitro*/*in vivo*. Several methods of encapsulation have been used to localize the delivery of the therapeutic agents. **Table 1** contains a comprehensive list of therapeutic agents, encapsulation strategies used, and the expected biological responses from their use. The effects of these therapeutic agents on preadipocyte differentiation and adipose tissue have been described further in illustration (**Figure 1**). FGF-2 is a member of fibroblast growth factors and most widely used angiogenic factor to stimulate blood vessel infiltration and subsequent de novo adipogenesis (Lu et al., 2014; Jiang et al., 2015; Zhang et al., 2016). Insulin binds to IGF-1 receptors to mimic most of the biological effects of IGF-1 (Lu et al., 2014). IGF-1 and insulin activates phosphorylation of cAMP response element binding protein (CREB) in preadipocytes to induce differentiation while also regulating triglyceride synthesis through sterol regulatory element-binding protein-1c (SREBP1c) (Widberg et al., 2009; Lu et al., 2014). Vascular endothelial growth factor (VEGF) is a major regulator of vasculogenesis and angiogenesis, serving as a mitogen for vascular endothelial cells (EC) along with several other roles (Chung et al., 2012). Pioglitazone hydrochloride (P-zone) promotes peroxisome proliferator activated receptor-γ (PPAR-γ) mRNA expression in preadipocytes, a critical step in adipogenesis, as well as increases mRNA levels of insulin responsive glucose transporter (GLUT4) (Lu et al., 2014; Yazawa et al., 2015). It is believed that FGF-1 increases expression of PPAR-γ and members of the CCAAT enhancer binding protein (C/EBP) family of transcription factors to prime preadipocytes for proliferation and differentiation (Hutley et al., 2004; Widberg et al., 2009). Dexamethasone (Dex) targets activating transcription factor 4 (ATF4) to initiate adipocyte differentiation in preadipocytes (Widberg et al., 2009). Direct comparisons of these additives in the appropriate animal model as well as their feasibility for commercial production are imperative for developing a clinically relevant adipogenic biomaterial.

**Table 1:** Therapeutic agents for adipose tissue defects.

Drugs/biomolecules	Methods of delivery	Cellular response	Reference
Dexamethasone	Double-emulsion/solvent extraction	Down-regulate expression of preadipocyte factor-1 (pref-1)	Rubin et al., 2009; Sun et al., 2013; Fan et al., 2015; Jia et al., 2015; Kelmendi-Doko et al., 2017
Insulin	Double-emulsion/solvent extraction; photocured to gelatin	stimulates glucose uptake, lipogenesis, and inhibits lipolysis through IGF-1	Masuda et al., 2004; Rubin et al., 2009; Tan et al., 2010; Kim et al., 2016
Insulin-like growth factor-1 (IGF-1)	Photocured to gelatin	Activates AKT and MAPK signaling pathways	Masuda et al., 2004; Kim et al., 2016
Basic fibroblast growth factor-2 (FGF-2)	Mixed heparinized protein; photocured to gelatin; hydrogel supplement; double emulsion	upregulates mitotic genes	Kawaguchi et al., 1998; Kimura et al., 2003; Masuda et al., 2004; Zhang et al., 2016
Pioglitazone hydrochloride (P-zone)	Saline gel mixture	Ligand for PPAR-γ; increase GLUT4 expression	Yazawa et al., 2015
Vascular endothelial growth factor (VEGF)	Double-emulsion/solvent extraction	Mitogen for vascular EC; increase EC migration, regulate microvascular permeability and vasodilation	Zhu et al., 2010; Chung et al., 2012; Topcu et al., 2012
Fibroblast growth factor-1 (FGF-1)	Heparin incubation	increase PPAR-γ expression	Widberg et al., 2009; Moya et al., 2010

*The effects of therapeutic agents on preadipocytes, vascular cells, and adipocytes have been exploited in numerous studies to enhance the functional behavior of biomaterials and autologous fat grafts*.

## Autologous Adipose Graft Implantation and Enhancement Strategies

**FIGURE 2:**
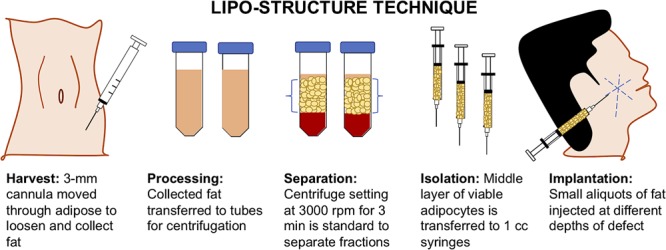
Lipo-structure technique for autologous fat grafting (AFG). Lipo-structure technique is a frequently used procedure for filling an adipose defect. The technique is performed in the operating room by plastic surgeons as the gold standard.

Depending on the clinical indication, the standard of care for adipose tissue repair and regeneration may vary as well as their outcomes. For cases such as breast reconstruction, lipoatrophy, scleroderma, face rejuvenation, gluteal augmentation, cranio-maxilla-facial deformities, etc., the gold standard of care is an AFG (Monfort and Izeta, 2012). AFG is a safe, resourceful, and minimally invasive option which allows the patient’s body to provide its own non-immunogenic, compatible biomaterial (Hong et al., 2010; Gir et al., 2012a; Agha et al., 2013). The liposuction technique and fat processing of AFG has significantly evolved over the past century with the advancement of technologies used to perform the procedure and examination of studies to optimize graft survival. As seen in **Figure 2**, many plastic surgeons have adopted the “lipo-structure technique” or Coleman technique for the microinjection of fat particles to minimize fat graft loss. Using a Coleman microcannula, light negative pressure is created by drawing the plunger of a 10-ml syringe connected to a 3-mm cannula when introduced in the subcutaneous cavity per a small incision. The cannula is moved through the adipose section manually, loosening the tissue, and collecting the fat into the syringe. For processing, the collected fat tissue is transferred to 10 mL tubes for centrifugation to separate into four layers: (a) oily fraction; (b) aqueous fraction; (c) a cell pellet; and (d) the purified fat flanked by the oily and aqueous fractions (Simonacci et al., 2016). Centrifugation at 3000 rpm for 3 min is the standard for separation of fractions, but there is literature that suggests slower speeds may reduce adipocyte disruption (Gir et al., 2012a). Following centrifugation, the middle layer of viable adipocytes is transferred to 1 cc syringes. Successful graft implantation is executed using blunt tip cannulas to create tunnels at insertion, with small aliquots of fat injected at different depths of the defect. Multiple passes are utilized to reach throughout the site of interest. Due to the unpredictable character of AFG, the majority of clinicians overcorrect volume deficiencies from 20 to 30% (Kaufman et al., 2007; Tabit et al., 2012).

While AFG is a widely utilizes treatment, there can be complications. Experienced physicians using the preferred technique may encounter unsatisfactory results such as fat necrosis, calcification, oil cyst formation, and hypertrophic scarring (Gir et al., 2012a; Philips et al., 2014; Nahabedian, 2015; Simonacci et al., 2016). Largo et al. (2014) reviewed several publications following the results of over 1400 patients. The studies containing volume retention data documented values varying from 55 to 82%. Other studies have reported resorption rates from 30 to 70% (Kaufman et al., 2007; Kurita et al., 2008). There are several mechanisms that contribute to the variability of outcomes from AFG. The trauma caused by liposuction can lead to apoptosis of adipocytes and cyst formation. The different degrees of blood supply and fluctuations in oxygen delivery of the recipient wound may range from adequate revascularization to insufficient revascularization and ischemia, apoptosis, and dedifferentiation of essential adipocytes. An accepted principle of fat grafting is that adipocytes within 2 mm of an arterial blood supply have greatest chance of survival (Tabit et al., 2012; Bourne et al., 2016).

New therapies which include both biomolecules and cells are now being studied to increase viability within AFG and consistently enhance retention. For example, Phipps et. al proposed the use of M2 macrophages to improve fat graft retention via proangiogenic factors such as VEGF and basic fibroblast growth factor-2 (FGF-2) (Phipps et al., 2015). Additionally, M2 macrophages provide matrix metalloproteinases required for endothelial sprouting and vascular network formation. Investigators injected fat grafts (150 mm^3^) with saline or M2 macrophages under the scalps of C57BL/6 mice. After 3 months, M2 supplemented grafts resulted in significantly higher mean volumes and exhibited an increase in vascular density compared to controls. Hong et al. (2010) explored the strategy of using supplemented transfer medium containing insulin and FGF-2 to enhance the viability of autologous fat grafts transplanted between the muscle and subcutaneous tissue of New Zealand white rabbits. Fat grafts were immersed for 5 min in either supplemented transfer medium or saline before transplantation. The insulin-supplemented fat grafts retained approximately 40% of original weight while fat grafts soaked in saline only retained ∼15% of original weight after 12 months. Additionally, groups treated with insulin and fibroblast growth factor showed larger number of mature adipocytes, and reduced cyst formation compared to the group treated with saline, at both 6 and 12 months after autologous fat transplantation.

Researchers have utilized biomaterials to encapsulate growth factors and pharmaceuticals aiming to develop clinically relevant tissue engineering therapies (Rubin et al., 2009; Chung et al., 2012; Topcu et al., 2012; Zhang et al., 2016). VEGF was encapsulated in calcium alginate microspheres to create a controlled release system and was evaluated in a Wistar rat model receiving fat grafts (Topcu et al., 2012). Rats in groups 1 and 2 received microspheres (MS) containing 2 μg/0.1 mL VEGF subdermally 21 days prior to fat grafting and mixed within fat graft, respectively. After 90 days, the fat graft control group lost over 50% of weight while groups receiving VEGF calcium alginate MS showed an increase in weight over 25%. Microvascular density results were also significantly higher in the VEGF microsphere pre-injection group and VEGF MS mixed with fat graft group. VEGF has also been encapsulated in poly(lactic-co-glycolic) (PLGA) MS as a potential therapy for transferred human lipoaspirate (Chung et al., 2012). Human lipoaspirate with and without PLGA VEGF MS were injected into the flanks of athymic mice and evaluated up to 6 weeks for vascularization and tissue survival. Significantly higher mean masses and volumes in the VEGF-loaded microsphere group were seen at 6 weeks. Additionally, there was IHC staining qualitatively displayed increased vascularization in the VEGF MS group at 6 weeks compared to 3 weeks results.

Rubin et al. utilized PLGA MS to encapsulate two adipogenic factors, dexamethasone (Dex) and insulin, as a method to stimulate adipogenesis in ASCs. Results showed that ASCs treated with dexamethasone and insulin microspheres contained a significantly higher number of adipocytes compared to control groups (Rubin et al., 2009). More recently, Kelmendi-Doko et al. (2014) utilized this same microsphere delivery system with encapsulated adipogenic factors to increase retention of transplanted adipose tissue. Dex and insulin were loaded into PLGA MS and mixed at various doses with human fat grafts to be injected subcutaneously into the dorsal aspect of athymic mice. After 5 weeks, volume analysis showed increased graft retention with use of Dex-loaded PLGA MS. Blood vessel density was also increased with the use of the adipogenic encapsulated MS. Kelmendi-Doko et al. (2017) extended the investigation of Dex MS in a double-walled (DW) formulation mixed into human fat grafts. Control human fat grafts explanted from athymic mice after 6 months demonstrated significant volume loss (i.e. ∼90%) while groups containing the DW Dex MS resulted in a mean volume loss of ∼33%, thus demonstrating the beneficial effects of a slow, controlled release delivery system.

In another study, Masuda et al. (2004) proposed the co-release of insulin with additional encapsulated drugs from styrenated, gelatin-based microspheres (SGM). Insulin, IGF-1, and FGF-2 were encapsulated to develop a drug delivery system that induces neovascularization and migration of endogenous preadipocytes (it is worth noting that ASCs can also be referred to as preadipocytes, due to their facile differentiation into adipocytes), followed by proliferation and differentiation into adipocytes. Subcutaneous lesion injections of SGMs in Wistar rats were evaluated after 6 weeks. The total lipid content extracted from the experimental group receiving FGF-2, insulin, and IGF-1 SGMs were significantly higher than groups receiving insulin SMGs alone, IGF-1 SGMs alone, and insulin/IGF-1 combined SGMs (Masuda et al., 2004).

Additional proposed therapies for improved retention and tissue restoration in AFG are the use of the stromal vascular fraction (SVF) and/or ASCs. SVF can be acquired during enzymatic collagenase digestion of excised human fat (Philips et al., 2012; Minteer et al., 2015). Clinically, SVF is attractive due to its isolation from tissue within ∼60–90 min. If the SVF is placed in culture, the ASCs adhere to the surface of tissue culture treated flask after 6–8 h of incubation at 37°C and 5% CO_2_. When SVF or ASC cells are mixed with the fat graft, the strategy is known as cell-assisted lipotransfer (CAL). Recently, Olenczak et al. (2017) reported the supplementation of fat grafts with SVF implanted into the scalp of a NOD-SCID mouse model. At 12 weeks, the mean volume of the human adipose tissue grafts with SVF did not show improved retention compared to control fat grafts alone. Histological results also showed no improvement in the frequency of cysts, fibrosis, or inflammation from the use of SVF. However, a human clinical trial recently conducted by Gontijo-de-Amorim et al. (2017) had contrasting results. Patients in the clinical trial had symmetric depletion of the facial volume and were randomly assigned a single-session of autologous fat transfer with adipose tissue alone (control) or adipose tissue combined with SVF cell pellet (enriched). Computed tomography results found that the enriched group had significantly better volume retention (9.6% volume loss vs. 24% in the control group) 12 months post-operation. Potential explanations for why these two studies had contrasting results include the lack of enzymatic digestion used in the clinical trial (difference in SVF isolation protocol) (Lockhart et al., 2017), the “xenograft vs. autograft” designation, and the amount of ASC content in each SVF.

While the previous two studies provide conflicting results, there is a substantial amount of evidence supporting the therapeutic use of ASCs to improve long-term graft survival and retention (Zhu et al., 2010; Philips et al., 2014). For example, Zhu et al. (2010) studied long-term graft retention in a mouse fat transplantation model with ASCs up to 9 months. Improved graft retention, higher capillary density, and reduction in cyst formation was observed in the fat transfer groups containing ASCs after 6 and 9 months. K∅lle et al. (2013) studied the efficacy of CAL in humans in the first randomized controlled study providing proof-of-principle for *ex vivo* expanded ASCs (Minteer et al., 2015). MRI results concluded the ASC-enriched fat grafts had significantly higher residual volumes compared to the control fat grafts with 80.9% vs. 16.3% of the initial volume 121 days from the surgical procedure. It is hypothesized that the angiogenic induction and minimal metabolic requirements by ASC contribute to the improved long-lasting graft results being observed. However, the field has yet to fully comprehend the molecular mechanisms of the role of ASCs in enhanced fat graft volume retention (Philips et al., 2014).

## Off-The-Shelf Biotechnologies and Scaffolds

Implant-based soft tissue reconstruction is arguably one of the most widely studied methods to restore contour at an adipose defect site (Schmauss et al., 2016). These implants are normally used in patients who want to avoid additional scars or cannot adequately donate adipose graft tissue. However, implant-based reconstructions are prone to develop local complications within 10 years, resulting in risks as high as 70% for additional surgery (Gabriel et al., 1997; Schmauss et al., 2016). Potential off-the-shelf biotechnologies such as tissue engineered scaffolds have the potential to greatly reduce the risk and complication of synthetic implants.

Scaffolds developed for permanently restoring soft tissue defects will need to consistently meet several standards while avoiding certain detrimental properties. Scaffolds should support cell attachment, migration, cell-cell interactions, cell proliferation, and cell differentiation (Brown et al., 2010; Bencherif et al., 2012; Londono and Badylak, 2015). The construct should be biocompatible with a controlled biodegradation rate proportional to newly developed adipose tissue integrating to the host environment. During the initial stages of tissue regrowth, scaffolds should provide mechanical support for cells and newly formed extracellular matrix (ECM). An interconnected network of pores should also be established to promote angiogenesis and nutrient diffusion (Teti, 1992; Keane et al., 2012). Some characteristics to avoid would include high resorption rates, foreign-body reaction, and capsular contracture. In response to the ideal adipose tissue scaffold, the host environment will eventually establish healing cues and have limited foreign-body reactions to the implant material (Aamodt and Grainger, 2016). This constructive remodeling response occurs with the help of selective components within the inflammatory, proliferation, and remodeling phase. The next several sections describe three different categories of biomaterials.

### Synthetic Biomaterial-Based Therapies

Synthetic biomaterials have the advantage of being tailored and specifically designed for adjustment of mechanical/chemical properties as well as degradation (Place et al., 2009). Synthetic materials such as poly(lactic acid) (PLA), poly(glycolic acid) (PGA), and poly(lactic-co-glycolic acid) (PLGA) have been extensively investigated and used for adipose tissue regeneration (Patrick et al., 1999, 2002; Patrick, 2001; Place et al., 2009; Itoi et al., 2010; Dhandayuthapani et al., 2011; Sharma et al., 2015; Elamparithi et al., 2016). These polymers degrade via hydrolysis and their degradability can be controlled by altering the molecular weight, crystallinity, and ratio of lactic to glycolic acid subunits (Place et al., 2009).

As ASCs and SVF are used in AFG retention, these cells have also been seeded within scaffold biomaterials as a therapy for adipose tissue engineering. Patrick et al. (2002) demonstrated *in vivo* tissue formation compared preadipocyte-seeded PLGA scaffolds to acellular PLGA scaffolds implanted subcutaneously on the dorsal aspect of Lewis rats for 12 months. Histological assessment of explanted constructs showed maximum adipose tissue formation at 2 months for both groups. However, as PLGA scaffolds degraded, adipose tissue volume also deceased with time. Long term maintenance of viable adipose tissue was not obtained using the preadipocyte-seeded PLGA scaffolds (Patrick et al., 1999, 2002). Cho et al. (2005), continued to analyze synthetic biomaterials to engineer adipose tissue using PLA reinforced PGA. The biodegradable PGA/PLA support scaffold was supplemented with fibrin matrix and seeded with human adipocytes prior to implantation into subcutaneous pockets of athymic mice. After 6 weeks, adipose tissue formation with scaffold-fibrin matrix containing preadipocytes indicated differentiation *in vivo*. Implanted scaffolds without preadipocytes in fibrin gel demonstrated limited adipocyte formation (Cho et al., 2005). The survival of adipose tissue past 6 weeks was not reported. It is possible that an immunocompetent animal model with autologous cells may provide additional information into the mechanism. These preliminary studies introduced the use of preadipocytes for adipose tissue formation. Several limitations such as time, feasibility, and reproducibility have been addressed by investigators. With the maximum adipose tissue formation being shown at 2 months, additional therapeutics are needed to provide stronger outcomes *in vivo* with synthetically based scaffolds. Synthetic materials possess several drawbacks when compared to their natural counterparts, particularly the absence of intrinsic surface ligands for cell attachment and a potential impact of their degradation products on cell function (Place et al., 2009; Dhandayuthapani et al., 2011). These limitations render the use of solely biomaterials for soft tissue repair quite challenging.

### Natural Biomaterial-Based Therapies

Collagen and hyaluronic acid are two of the most common naturally derived biomaterials for scaffolds in tissue engineering. The enzymatically degradable glycosaminoglycan, hyaluronic acid (HA), consists of multiple repeating disaccharide units of N-acetyl-D-glucosamine and D-glucuronic acid. HA is a major component of the extracellular matrix due to its prominent role in cellular behavior, mechanical support, nutrient diffusion, high water retention, and intrinsic swelling property. HA-based scaffolds are currently available on the market for various surgical procedures (Stillaert et al., 2008; Itoi et al., 2010; Cohen et al., 2013).

The potential of HA as an off-the shelf biomaterial for adipose tissue growth has been studied by several research groups. Tan, et al. developed an injectable thermosensitive HA gel that confirmed in situ gel formation after subcutaneous injection into athymic mice up to 5 days (Tan et al., 2009). However, HA has been known to undergo rapid absorption *in vivo* (Hemmrich et al., 2008; Okabe et al., 2009). A composite device involving an HA-based (HYAFF^®^11) pre-adipocyte seeded scaffold implanted in subcutaneous pockets of human volunteers, using a 1 cm median sub-umbilical skin incision, up to 16 weeks, has been reported (Stillaert et al., 2008). Histological analysis results indicated that the HA-based scaffold with and without preadipocyte seeding lacked evidence of any mature adipocytes at 16 weeks. In summary, the results confirm the current proposed composite scaffolds are not inductive toward adipose tissue formation with deficient angiogenic infiltration (Itoi et al., 2010).

Enhancing the adipogenic properties of hydrogels may be achieved by combining drugs or cytokines. For example, Fan et al. (2015) attempted to improve adipose tissue formation in HA hydrogels through aqueous Diels–Alder chemistry. The HA hydrogel was functionalized for a sustained release of dexamethasone over a 2-week time period. *In vitro* studies conveyed significant increases of the ASC population in groups containing the functionalized Dex-HA hydrogel compared HA gels after 14 days. The same mechanism of drug release was used in magnetic HA nanospheres capable of delivering dexamethasone controlled by an external magnetic field (Jia et al., 2015). *In vitro* ASC culture results confirmed an increase in cell viability and activity with magnetic HA nanospheres with Dex under a magnetic field. Controlled drug/biomolecule delivery continues to be an evolving asset in tissue restorative therapies.

Collagen is a naturally derived ECM protein that provides biodegradability, biocompatibility, and weak antigenicity. Several collagenous based biomaterials have been studied due to their favorable characteristics, long history of safety, and FDA-approval. One of the earliest studies of collagen and FGF-2 therapy reported the use of reconstituted basement membrane extracellular matrix, Matrigel^TM^, to deliver FGF-2 subcutaneously in BALB/c nude mice at several concentrations (Kawaguchi et al., 1998). Experimental groups receiving Matrigel^TM^ supplemented with FGF-2 at 10, 100, and 1000 ng/ml, saw neovascularization within 7 days, followed by migration of endogenous preadipocytes. A visible fat pad was observed between 1 and 2 weeks and was maintained up to 10 weeks (Kawaguchi et al., 1998). An alternative to free FGF-2 delivery has been investigated in gelatin microspheres containing 1 μg incorporated in the Matrigel^TM^ scaffold along with ASCs (Kimura et al., 2003). Using a BALB/c nude mouse model, a significant difference in adipose tissue formation was discovered using gelatin microsphere encapsulated FGF-2 compared to free FGF-2 in the collagen sponge subcutaneous implant. Additionally, the most adipose tissue was formed using 2.5 × 10^4^ ASCs/cm^2^ of the implant site compared to 1.25 × 10^4^ and 5 × 10^3^cells/cm^2^ (Kimura et al., 2003).

Matrigel^TM^ has also been investigated with alginate microbeads containing fibroblast growth factor-1 (FGF-1) to induce neovascularization and adipogenesis in a rat vascular pedicle model of adipose tissue engineering (material of interest deposited in silicone tube with vasculature wrapped in local fat pad) (Moya et al., 2010). Vessel density and area of adipose tissue was measured are 6 weeks. Compared to a bolus injection of FGF-1 and empty alginate microbeads, Matrigel^TM^ containing 2.5 μg/ml of FGF-1 in microbeads experienced higher rates of vessel density. However, no difference in adipose tissue formation was observed between the bolus injection group and FGF-1 microbeads group at 2.5 μg/ml dose (Moya et al., 2010).

Porous collagenous microbeads (CultiSphers; Sigma, St. Louis, MO, United States) have been evaluated as an injectable cell delivery scaffold for ASCs (Rubin et al., 2007). *In vitro* studies using a spinner flask showed ASCs in culture attach and seed into microbeads at a high capacity. The ability to proliferate and differentiate was also confirmed with clear potential in culture up to 49 and 21 days, respectively (Rubin et al., 2007). An assessment of cell viability post-injection and tissue growth *in vivo* are the logical next steps to advance this technology for adipose tissue regeneration. Since these early studies, there have been multiple *in vivo* studies examining collagen in adipose tissue engineering (von Heimburg et al., 2001; Kimura et al., 2003; Vashi et al., 2006; Itoi et al., 2010; Cherubino et al., 2016).

While the majority of studies with collagen consisted of bovine collagen, salmon collagen has also been evaluated as a scaffold for its ligand protein interactions. This protein biomaterial was prepared with and without pioglitazone, a known inducer of adipogenesis via PPAR-γ (Yazawa et al., 2015). Using a C3H/He/N mouse model, biomaterials were injected subcutaneously and analyzed at 1 and 4 weeks. Histological results showed mature adipocyte growth substituting the pioglitazone collagen after 4 weeks compared to minimal tissue growth in the control group without pioglitazone.

While scaffold performance of collagen and HA has been analyzed individually, the combined form of these natural biomaterials have implications on the future use of multi-component scaffold. Collagen (type I, derived from bovine Achilles) and HA (derived from bovine vitreous humor) has been crosslinked to develop a three-dimensional scaffold that encourages adipose tissue development (Davidenko et al., 2010). Collagen-HA scaffolds were characterized, seeded with preadipocytes, and compared to collagen scaffolds. The results indicated that collagen-HA significantly increased gene expression of adipsin, an enzyme involved in lipid metabolism predominately in mature adipocytes (Lu et al., 2014), and noticeably higher levels of PPAR-γ. The inclusion of HA in collagen scaffolds may aid adipogenesis by hastening cell-contacted growth arrest prior to adipogenic conversion (Davidenko et al., 2010). Preadipocytes seeded on collagen coated with elastin has also been recognized as a potential scaffold with the ability to encourage cell proliferation, infiltration, and adhesion (Keck et al., 2011).

Gelatin, a partially degraded product of collagen, contains amino acid sequences, which can enhance cell attachment (Chang et al., 2013). Gelatin has also been combined with hyaluronic acid as a complementary ingredient for cryogel scaffolds. Gelatin-HA cryogel scaffolds were seeded with porcine ASCs and implanted into the subcutaneous pocket of two separate animal models: murine and porcine. Acellular cryogels were also implanted for comparison. The relative gene expression of adipocyte-specific genes (PPAR-γ, LPL, aP2, and leptin) were significantly greater in the seeded gelatin-HA scaffolds than acellular scaffolds at weeks 2, 4, and 8 in both animal models. It is important to note that both seeded and acellular scaffolds exhibited positive CD31 staining at 8 weeks showing how adequate porosity sufficiently provided space for vascularization in the acellular scaffold implants, a characteristic many other acellular scaffolds failed to demonstrate (Chang et al., 2013).

Chitosan (CS) is another biodegradable natural material currently being used in tissue engineering applications (Tan et al., 2010; Wang et al., 2013a,b). The biocompatible characteristics of cell adhesion and growth were determined using crosslinked CS with poly (L-glutamic acid) to mimic natural ECM (Wang et al., 2013a). A porous scaffold was developed via lyophilization for evaluation of engineered adipose tissue in SCID mice. Acellular and ASC-seeded scaffolds were implanted. After 6 weeks, scaffolds remain intact with vascularization reported in the cell-seeded scaffolds. Cell-seeded poly (L-glutamic acid)/CS scaffolds retained 90% volume and Oil red O staining indicated adipose tissue formation within the implant (Wang et al., 2013a). Minimal adipose tissue was formed within acellular implants. CS has also been crosslinked with HA to produce an injectable hydrogel scaffold capable of delivering dexamethasone for adipose tissue growth (Sun et al., 2013). Test results in ASC culture revealed dexamethasone as an important factor in hydrogel performance with increased proliferation and cell adhesion.

Silk is another widely examined biomaterial for soft tissue repair. Silk is a naturally occurring biocompatible protein with tunable mechanical strength, adjustable degradation rate, and low inflammatory and immunogenic responses (Bellas et al., 2015; Abbott et al., 2016). In reference to the application of silk to soft tissue regeneration, Bellas et al. (2015) evaluated silk at three concentrations in the form of an injectable foam scaffold. Silk foams supported *in vitro* ASC survival and migration over a 10-day period. Subcutaneous injections of silk scaffolds in Sprague-Dawley rats demonstrated significant degradation after 90 days with new tissue formation, and cell-seeding ASCs into the silk scaffold may have contributed to new adipose tissue growth *in vivo*.

Overall, the use of natural-biomaterial based therapies has led to considerably positive results both *in vitro* and *in vivo*. The use of ASCs for *in vivo* injection studies showed significance on several examples. More comparison studies of natural biomaterials containing ASCs with different therapeutic agents *in vivo* would be immensely revealing to see if a specific grouping is ideal.

### Extracellular Matrix (ECM) Biomaterial-Based Therapies

An ECM scaffold-based approach to regeneration will elicit an immediate host response distinctly different from other scaffold materials after implantation *in vivo* due to their surface topologies and ligand landscapes (Brown et al., 2012b). Cellular infiltrate consisting of polymorphonuclear leukocytes and mononuclear cells occurs within min. After 72 h, most infiltrating cells are entirely mononuclear, specifically of the pro-inflammatory macrophage phenotype (M1), with early signs of neovascularization. For 2 weeks, the mononuclear cell population continues to infiltrate the ECM scaffold, including macrophages for apoptotic neutrophil removal, angiogenesis dramatically increases, and degradation of scaffold is advanced as newly formed host ECM is deposited. By day 14, a reduced mononuclear cell population consisting mostly of immunoregulatory macrophage phenotype (M2) resides and site specific parenchymal cells appear to begin the final phase of constructive remodeling along with circulating, marrow derived progenitor cells. By day 35, there should be minimal to no scaffold present and a dense, highly organized connective tissue present (Badylak, 2002, 2004; Brown et al., 2012a,b).

Despite a multitude of publications investigating the use of synthetic and natural materials for adipose tissue engineering, there remains a clinical need to have an off-the-shelf biomaterial for adipose restoration. Recent advances in decellularization have provided researchers an alternative material to employ in all areas of tissue engineering. Ideally, decellularization is designed to remove the immunogenic material from donated biological sources while retaining as many constitutive components as possible. ECM can be derived from a specific tissue source to exploit its unique assortment of endogenous factors to restore its respective milieu. Many endogenous factors such as VEGF, transforming growth factor (TGF) beta 1, basic fibroblast growth factor, placenta growth factor and insulin-like growth factor 1 affecting angiogenesis have been identified in adipose tissues (Choi et al., 2014). Decellularized adipose tissue (DAT) has gained significance in the tissue regeneration field as scaffold material in the form of hydrogels, sponges, injectable hydrated material, and fibers constructed from adipose ECM.

Several groups have validated DAT hydrogel from human and porcine as a support structure for cellular activity *in vitro* (Uriel et al., 2008; Choi et al., 2011, 2014; Young et al., 2011; Poon et al., 2013; Sano et al., 2014). The investigation of porcine adipose ECM utilized freeze-thaw cycles along with trypsin/EDTA to decellularize porcine tissue. Resultant porcine derived biomaterial demonstrated DNA content 99.5% less than native tissue and successful cellular infiltration *in vitro* over 8 days. However, the lack of animal research for adipose growth undermines its actual potential (Roehm et al., 2016). A pepsin digest was used to create an injectable hydrogel from dry milled human DAT; endogenous proteins like collagen I, III, and IV were present after processing (Young et al., 2011). Gelation of the hydrogel occurred with subcutaneous bolus injections in athymic mice. However, implants were excised 15 min later and further points in time were not investigated. In a later publication, this research was updated to include an ASC-seeded scaffolds with or without transglutaminase (TG), an angiogenic cross-linker (Adam Young et al., 2014). It was discovered that the addition of TG and ASCs significantly improved vascularization *in vivo* compared to the human DAT hydrogel. Oil red O staining of the scaffolds after 4 weeks *in vivo* also indicated superior adipogenesis in human DAT hydrogel containing TG and ASCs (Adam Young et al., 2014).

It has been hypothesized that adipose derived stem cells in combination with DAT would be therapeutic for stimulating natural soft tissue regeneration. Several groups have investigated this hypothesis including the fabrication of an injectable microparticle formulation in saline for engineered fat graft evaluation. Human ASCs (hASCs) were combined with human DAT to study adipose tissue engineering implanted subcutaneously in nude rats for up to 8 weeks compared to human fat graft and hASC injection. Vessel density confirmed by IHC staining showed that human DAT-ASCs grafts at 4 and 8 weeks contained less vessels compared to the fresh human fat grafts. H&E staining also confirmed remodeling of fresh graft and surrounding tissue resembling new adipose formation (Wang et al., 2013). In a cell culture comparison study, ASCs were induced in adipogenic media for 2 weeks and seeded onto various scaffolds (type I collagen sponge, PGA, and hyaluronic acid) prior to implantation into athymic mice. Compared to ASCs seeded in normal culture media in various scaffolds, the area of newly generated adipose tissue was significantly increased in the induced ASCs group. However, within the induced ASC group, only the type I collagen sponge displayed noticeable amounts of adipogenesis after 8 weeks. Induced ASCs in PGA and hyaluronic acid gel displayed minimal adipocyte growth (Itoi et al., 2010). In summary, the results confirm the current proposed composite scaffolds are not inductive toward adipose tissue formation with deficient angiogenic penetration (Itoi et al., 2010).

As a strategy to increase clinical relevance, Han et al. assessed allogenic rat ASC-seeded DAT scaffolds implanted subcutaneously in an immunocompetent Wistar rat model. Cells were seeded 72 h prior to implantation (Han et al., 2015). Compared to non-seeded scaffolds, significant remodeling into mature adipocytes was observed in the ASC-seeded groups at 8 and 12 weeks. Adipogenic marker expression in surrounding macrophages confirmed these results as well as correlated increases blood vessel diameter at 12 weeks. The scaffold platform proposed in this study provides invaluable preclinical data toward clinical trials (Han et al., 2015).

Specific ECM and cellular parameters can be adjusted to influence cellular behavior. For example, the effect of DAT particle size and cell density on ASC proliferation and differentiation was assessed in a composite methacrylate chondroitin sulfate (MSC)-DAT hydrogel. At a cellular density of 2500 cells/μl of MSC-DAT hydrogel, proliferation of human ASCs was significantly higher than using DAT of large particle size (∼280 μm) compared to the small particle size group (∼40 μm). However, at an ASC concentration of 5000 cells/μl of hydrogel, the small particle size group displayed a significantly outperformed all groups with the greatest amount of adipogenesis, identified by glycerol-3-phosphate dehydrogenase activity and laminin production (Brown et al., 2015).

In an attempt to enhance fat graft volume retention, FGF-2 was loaded into an injectable decellularized adipose matrix (mouse-derived tissue) to be investigated as a potential soft-tissue replacement for reconstructive surgery (Zhang et al., 2016). A 12-week animal study compared to performance of FGF-2-Loaded DAT to PBS hydrated DAT in the form of subcutaneous injections. Results indicated significantly more adipose neotissue formation and higher volume retention at 6 weeks in the FGF-2 group. Adipocyte quantifications at 12 weeks for the FGF-2-loaded group were also comparable to endogenous mouse adipose (Zhang et al., 2016).

The use of decellularized ECM from tissues other than adipose have also been examined for soft tissue repair. For example, PLGA microspheres containing FGF-2 were combined with decellularized small intestinal submucosa (SIS) particulates and ASCs in an athymic mouse subcutaneous model for adipose tissue growth (Marra et al., 2008). Initially, ASCs were cultured and allowed to proliferate on SIS particulate before injection. Following the SIS injection, mice received either 1 ng of free FGF-2, 1 ng dose of FGF-2 in PLGA microspheres, or no FGF-2. Blood vessel quantification was significantly higher in mice receiving FGF-2 microspheres (Marra et al., 2008).

## Animal Models for Adipose Tissue Repair

A variety of animals has been used in adipose tissue research, each serving a specific purpose. Athymic mice are frequently used to assess volume retention of human fat grafts under various experimental conditions (microspheres) as well as the growth/differentiation of human ASCs in scaffolds within a living system. Athymic mice provide the advantage of physiological conditions for a multi-week study without having to match cell-to-animal species. Substantial information can be obtained from these studies, but it is distant from clinical application in terms of size, function, and immune health.

The use of an immunocompetent animal such as the Wistar rat is also common. The use of allogenic cells, autologous fat, and multi-component scaffolds have been explored and evaluated in the Wistar rat model. Materials are normally injected as a bolus subcutaneously with studies lasting up to 12 weeks. An animal model with a healthy immune system allow investigators to fully consider material degradation and immune responses related to adipose tissue formation. Adipose volume retention has been reported in this animal model at 12 weeks (Topcu et al., 2012), providing significant data with some clinical relevance. However, adipose defect repair has yet to be implemented in the Wistar rat, which would more closely reflect the clinical need.

It would be useful to identify an animal model that reflects the nature of an adipose defect where subcutaneous tissue is removed and a potential scaffold is implanted to demonstrate inherent tissue regeneration. Some animals that could be appropriate for this model would be the rabbit, guinea pig, or pig. These animal models would also be appropriate to assess different size defects to identify any size limitations for a potential regenerative scaffold. Once a desirable biomaterial has been established, investigators must be able to approximately quantify the amount of tissue formation per concentration of drug and/or biological factors within scaffold of choice on top of the optimal cell concentration. These results and parameters are critical for translation of these biotechnologies as well as their appropriate application to an array of unique adipose defect cases in the clinic.

## Conclusion

The investigation of various therapies for sustained adipose tissue regeneration has advanced significantly since AFG was introduced by Neuber (1893) and Tabit et al. (2012). Early on, clinicians saw the limitations of AFG and sought ways to work with researchers to improve patient outcomes. While AFG enhancement strategies have significantly improved, investigators continue to seek the benefits of establishing off-the-shelf biotechnologies for adipose tissue repair. Some of the earliest studies exploited the principles of synthetic, man-made materials for constructing implants and scaffolds that were temporary solutions to the lack of functional adipose tissue. Naturally derived biomaterials and decellularized proteins are currently prevalent in the research area as the base component of biologically active therapeutic strategies. From the beginning, ASCs have shown an important role being that these undifferentiated precursor cells provide the appropriate population for neovascularization and adipocyte formation. It is anticipated that biodegradable biomaterials will continue to dominate the future of tissue engineering, specifically adipose tissue. These complex, multi-component scaffolds require several fabrication steps to develop each component. With this degree of complexity involved in fabricating each scaffold, consistency and standardized batch testing will be of immense importance. Although these factors are in the preclinical stage, the addition of personnel with controlled drug delivery as well as manufacturing experience in the research lab can give investigators a clear advantage for the implementation of their complex biomaterial technology.

## Author Contributions

CM summarized the literature, wrote the review, drew the figure, and constructed the table. CI summarized literature related to biomolecules for adipose tissue repair. CY and KM provided critical comments on content.

## Conflict of Interest Statement

The authors declare that the research was conducted in the absence of any commercial or financial relationships that could be construed as a potential conflict of interest.
